# Combined Labelled and Label-free SERS Probes for Triplex Three-dimensional Cellular Imaging

**DOI:** 10.1038/srep19173

**Published:** 2016-01-19

**Authors:** Yong Chen, Xiangru Bai, Le Su, Zhanwei Du, Aiguo Shen, Arnulf Materny, Jiming Hu

**Affiliations:** 1 Key Laboratory of Analytical Chemistry for Biology and Medicine, Ministry of Education, College of Chemistry and Molecular Sciences, Wuhan University, Wuhan 430072 (China); 2 College of Computer Science and Technology, Jilin University, Changchun 130012 (China); 3 Department of Physics & Earth Sciences, Focus Area Health, Jacobs University Bremen, Campus Ring 1, 28759 Bremen (Germany)

## Abstract

Cells are complex chemical systems, where the molecular composition at different cellular locations and specific intracellular chemical interactions determine the biological function. An *in-situ* nondestructive characterization of the complicated chemical processes (like e.g. apoptosis) is the goal of our study. Here, we present the results of simultaneous and three-dimensional imaging of double organelles (nucleus and membrane) in single HeLa cells by means of either labelled or label-free surface-enhanced Raman spectroscopy (SERS). This combination of imaging with and without labels is not possible when using fluorescence microscopy. The SERS technique is used for a stereoscopic description of the intrinsic chemical nature of nuclei and the precise localization of folate (FA) and luteinizing hormone-releasing hormone (LHRH) on the membrane under highly confocal conditions. We also report on the time-dependent changes of cell nuclei as well as membrane receptor proteins during apoptosis analyzed by statistical multivariate methods. The multiplex three-dimensional SERS imaging technique allows for both temporal (real time) and spatial (multiple organelles and molecules in three-dimensional space) live-cell imaging and therefore provides a new and attractive 2D/3D tracing method in biomedicine on subcellular level.

Advances in the development of fluorescent probe technology have greatly facilitated label-imaging analysis of cellular functions^1^,^2^,^3^. However, the existing fluorescent probes, e.g., organic dyes, silica nanoparticles, and quantum dots, only allow for the investigation of the interactions among a limited number of molecules (≤10)^4^ because the emission peak of the fluorescent probes is too wide (typically 50 nm) to enable the differentiation of more overlapping emissions. Techniques that allow for a non-destructive and multidimensional approach for gaining simultaneously complex chemical information including multiplex molecular compositions, locations, and molecular interactions in living cells would be preferable.

Recently, nanoparticles (NPs) used as substrate for surface-enhanced Raman scattering (SERS) have attracted considerable attention as an emerging class of biolabels in cellular imaging^5^,^6^,^7^,^8^. One reason for this is that SERS provides a 10^6^–10^14^ fold enhancement of the Raman signal intensity, which is sufficient even for single molecule detection^9^. Secondly, the extremely short scattering times of the Raman process prevent photo-bleaching, energy transfer, or quenching of reporters in the excited state^10^, resulting in a high photostability of the SERS probes. Thirdly, an optimal contrast can be achieved by using red to near-infrared (NIR) excitation to minimize the disturbing autofluorescence of cells and tissues, making SERS an important tool for noninvasive imaging in living subjects^11^. This use of excitation wavelenghts, which are not resonant with electronic absorption transitions of the samples, additionally contributes to a reduced disturbance of the biological system. And finally, SERS tags yield much narrower peaks (typically <2 nm) in the resulting spectral response and thus offer the potential to access an increased number (about 10–100) of unique optical ‘signatures’ by varying the Raman reporter molecules, which make them ideally suited for multiplex detection or multicolour imaging^4^,^12^,^13^,^14^,^15^,^16^,^17^. Except for label imaging, a potentially label-free imaging method based on strong SERS signals from the native chemical constituents of a cell was developed by incorporating colloidal noble metal (Au or Ag) NPs into a cell, localizing them on subcellular organelles or bio-macromolecules. Therefore, unlike fluorescence microscopy, this type of SERS microscopy provides a tool for the sensitive and structurally selective detection of native chemicals inside a cell and their intracellular distribution, suggesting many applications in biomedical research^18^,^19^. Furthermore, the high vertical resolution (~1 μm) of a confocal Raman system yields an excellent stereoscopic description of a whole cell performing a layer-by-layer scan. It can be expected that label and label-free SERS cellular imaging capable of monitoring simultaneous subcellular events during cellular processes (e.g., apoptosis) will help to enhance the knowledge about the basic mechanisms of the cellular behaviour. However, up to date, the goal using this technique to simultaneously and stereoscopically describe several subcellular organelles and biomolecules in single cells, and more importantly, to directly obtain the intrinsic chemical information of certain subcellular organelles has not been achieved to a satisfying extent.

Here, we report the development of a new SERS strategy for triplex three-dimensional SERS imaging of a live cell, which allows for simultaneous SERS imaging of nucleus and membrane with high spatial resolution by means of confocal Raman microscopy. We show that representative and distinctly different intrinsic Raman signals of biomolecules from both membrane and nuclei can be selectively enhanced within single cells through membrane and nuclear-targeting label-free SERS probes, respectively. The Raman signals of two kinds of membrane targeting SERS probes and one nuclear-targeting SERS probe can be obtained from the same cell in three dimensions. Furthermore, the two types of SERS probes (both labelled and label-free) will be used for the detection of apoptotic cells on single-cell level. Finally, we will demonstrate that the combination of labelled and label-free SERS probes enable us to capture the dynamic chemical information of nuclei and at the same time, to display the real-time location of the FA receptors (FA-Rs) and the receptors for LHRH (LHRH-Rs) on the membrane with very good height resolution. Our results demonstrate that our novel SERS-based single cell analysis approach offers powerful strategies for multiplex three-dimensional cellular imaging.

## Results and Discussion

For the preparation of SERS probes, spherically shaped gold nanoparticles (AuNPs) were chosen as SERS-active material for SERS imaging. First, citrate-stabilized AuNPs of 40 nm diameter were prepared by using sodium citrate reduction. Then, 10 mL of AuNPs solution were incubated in the presence of 20 μL of 1 mM 4-mercaptobenzoic acid (MBA) ethanol solution for 12 h at room temperature, attaching the Raman-dye (MBA) to the AuNPs surface through an Au-S chemical bond. The mixture was centrifuged (4000 rpm, 15 min) to remove excess MBA before it was dispersed in 10 mL of deionized water (DW). Afterwards, 1 mL of an aqueous solution (0.1%, w/w) of poly-allylamine (PAH, MW = 17000) was added and the mixture was shaken gently overnight at room temperature to reduce aggregation and generate –NH_2_ groups on the particles’ surfaces. This was advantageous for the following modification with the -COOH group by EDC (1-Ethyl-3-(3-dimethylaminopropyl) carbodiimide) reactions or with the –NH_2_ group by glutaric dialdehyde^20^, and a large number of dye-molecules could be encapsulated in the PAH shell through electrostatic interactions between the molecules and the surfaces of the AuNPs (thiolation was insignificant). Finally, in order to promote the localization of the AuNPs at the cell nucleus, nuclear localization signal (NLS, GGVKRKKKPGGE) peptides were conjugated to the AuNPs by generating a stable amide with PAH-polymer layer^21^,^22^,^23^. By this, a simple, universal synthesis method of SERS AuNPs with nuclear-targeting ability could be realized.

**Figure 1:**
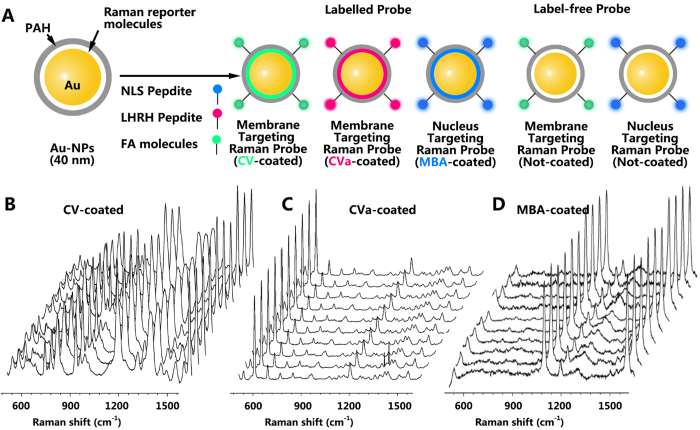
(**A**) Scheme for the PAHylation and peptide/micromolecule modifications on the Raman dye-coated Au-NPs: CV-coated Au-NPs were modified with PAH and FA for targeting the membrane; CVa-coated Au-NPs were modified with PAH and LHRH peptide for also targeting the membrane; MBA-coated Au-NPs were modified with PAH and NLS peptide for targeting the nucleus. (**B–D**) Representative Raman spectra of CV-coated AuNPs (**B**), CVa-coated AuNPs (**C**), and MBA-coated AuNPs (**D**) obtained from particle solution with an incident laser power of 3.0 mW and 1 s exposure time per spectrum.

In order to accomplish multi-targeting SERS imaging of cells, we have selected two more Raman-dyes (crystal violet (CV) and cresyl violet acetate (CVa)) as SERS reporters. These Raman-dyes were successfully encapsulated in the PAH shell through physical adsorption between the molecules and the AuNPs surfaces. After PAHylation of the dye-coated AuNPs, the AuNPs were subsequently modified with luteinizing hormone-releasing hormone (LHRH) and folate (FA) for membrane-targeting^24^,^25^. and NLS for nucleus-targeting via amide covalent linkages (Fig. 1A). In order to obtain the Raman spectra of biomolecules from the cell membrane and the nucleus without notable influence arising from the Raman reporters, the label-free targeting SERS AuNPs were designed as shown in Fig. 1A. The successful conjugation to PAH, NLS or FA, LHRH ligands was confirmed through an approx. 3 nm thick low-contrast layer surrounding the metal in the TEM images and a slight red shift of the surface plasmon resonance frequency compared to citrate-AuNPs as shown in Figure S1. The change of the plasmon frequency due to the citrate layer was destroyed by the conjugation. The CV-coated, CVa-coated, and MBA-coated AuNPs solutions displayed intense and uniform Raman response as shown in panels B to D of Fig. 1, respectively. This is an important property of the Raman probes for cellular imaging application^26^. It should be noted that the relatively low SERS intensity of label-free AuNPs revealed that NLS peptide and PAH ligands do not cause a significant Raman response during the detection of the intrinsic SERS signals from the cell components (Figure S2, black line). In this test, each Raman spectrum was obtained with an excitation laser at 632.8 nm (1 s and 3.0 mW incident laser power).

We selected the human cervix carcinoma cell line (HeLa) for single cell imaging due to the well-established interactions between nanoparticle with targeting ligands and HeLa cell^19^,^20^,^21^,^22^,^23^,^24^,^25^,^26^,^27^. In this work, SERS imaging of HeLa cells incubated with the AuNPs were performed using a Jobin-Yvon micro Raman system with a 50x objective lens. High-resolution SERS images could be achieved by adjusting the pinhole to less than 75 μm. In order to confirm the feasibility, the dye-coated AuNPs were incubated with the living HeLa cells. Suitable interaction times between each kind of AuNPs and the HeLa cells were determined by multiple imaging at the different incubation times. Through the high-resolution SERS images shown in Figure S3A, we found that there were a few MBA-coated AuNPs entering into HeLa cells after 2 h of incubation. With the increase of incubation time, as we expected, more AuNPs were detected in the cells. When the incubation time was increased to 12 h, a nuclear image was formed by the MBA-coated AuNPs. The images based on the CV-coated AuNPs at different incubation times are shown in Figure S3B. According to the predictions, the CV-coated AuNPs should be mainly distributed in a circle around the cells, and no distribution within the cells should occur. Interestingly, an ideal “circle” in the membrane-targeting experiment was obtained after 2 h of incubation; subsequent tests after longer incubation times show a deviation from the ideal circular distribution. It should be noted that the experimental parameters related to the confocality (i.e., 75 μm pinhole, depth in the z-direction) were exactly the same in all experiments. The reason for the decreasing specificity of the membrane-targeting AuNPs to the membrane beyond 2 hours of incubation is the endocytosis of the cells. In the process of cell metabolism, usually the macromolecular substances cannot directly be transferred through the cell membrane. In order to allow the material transport into the cell, membrane-invaginated vesicles are locally formed. By this, part of the AuNPs enters the cells and a strong Raman signal can be detected with increasing observation time. From our experiments, we conclude that the optimum of incubation time for membrane- and nuclear-targeting is 2 h and 12 h, respectively.

**Figure 2:**
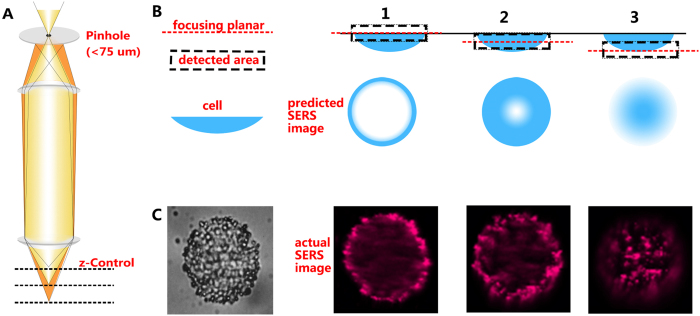
(**A**) The principle schematic drawing of the confocal microscope. (**B**) Schematic diagram of the SERS images on different axial position (z-direction). (**C**) High-resolution SERS images in taken step by step from different “z-slices” of the same cell by adjusting the focal plane in z-direction (scale bar ≡ 4 μm).

Cells are three-dimensional structures. Therefore, in standard microscopy with limited height resolution, the Raman image based on the membrane-targeting AuNPs should be a “disc” rather than a “circle”. The ring-like structure observed demonstrates the high axial resolution of the confocal Raman-microscope. Figure 2 shows the schematic drawing of the confocal microscope and the SERS image as a function of axial position (z-direction). Step-by-step 3D SERS images of the cell can be obtained by accessing the different “z-slices” scanning the focal plane along the z-direction. Images taken at different z-positions are displayed in Fig. 2. They clearly demonstrate that the confocal Raman spectrometer is well suited for 3D imaging^28^. The results of a more systematic study of the 3D SERS imaging capabilities of our technique, are shown in Figure S4. We have taken high-resolution SERS images in different “z-slices” of the same cell by varying the axial position of the focal point in intervals of 3 μm. Besides the Raman images, we have also taken dark and bright-field images. The distribution of the AuNPs in different “z-slices” can be nicely observed. These results also confirm the localization of membrane-targeting SERS AuNPs. The SERS images of the z-slices at 0, 3, and 6 μm position correspond to the schematic drawings 1–3 in Fig. 2B. The circlular shape of the Raman features discussed earlier is just a result of the confocal imaging of the cell close to its waist.

**Figure 3:**
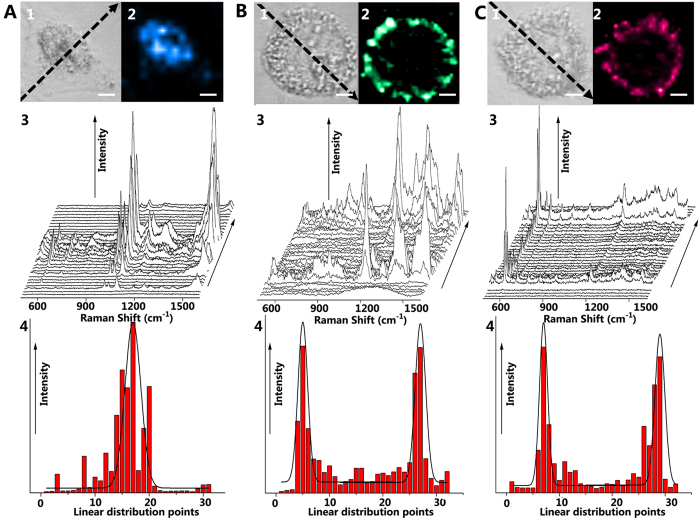
SERS imaging of a HeLa cell treated with MBA-coated AuNPs (A), CV-coated AuNPs (B), and CVa-coated AuNPs (C). (A1–C1) Bright-field images and (A2-C2) SERS images corresponding to the detected HeLa cell. (A3–C3) SERS spectra obtained from different positions within the cell via point by point detection (1 μm × 1 μm) along the dotted line in (A1–C1) with an incident laser power of 3.0 mW and 1 s exposure time per spectrum. (A4–C4) The histogram displays the Raman intensity at 1078 cm^−1^ (MBA-coated, A4), 1175 cm^−1^ (CV-coated, B4), and 595 cm^−1^ (CVa-coated, C4) obtained from the spectra in (A3–C3) along the dotted line in (A1–C1) (scale bar ≡ 4 μm).

**Figure 4:**
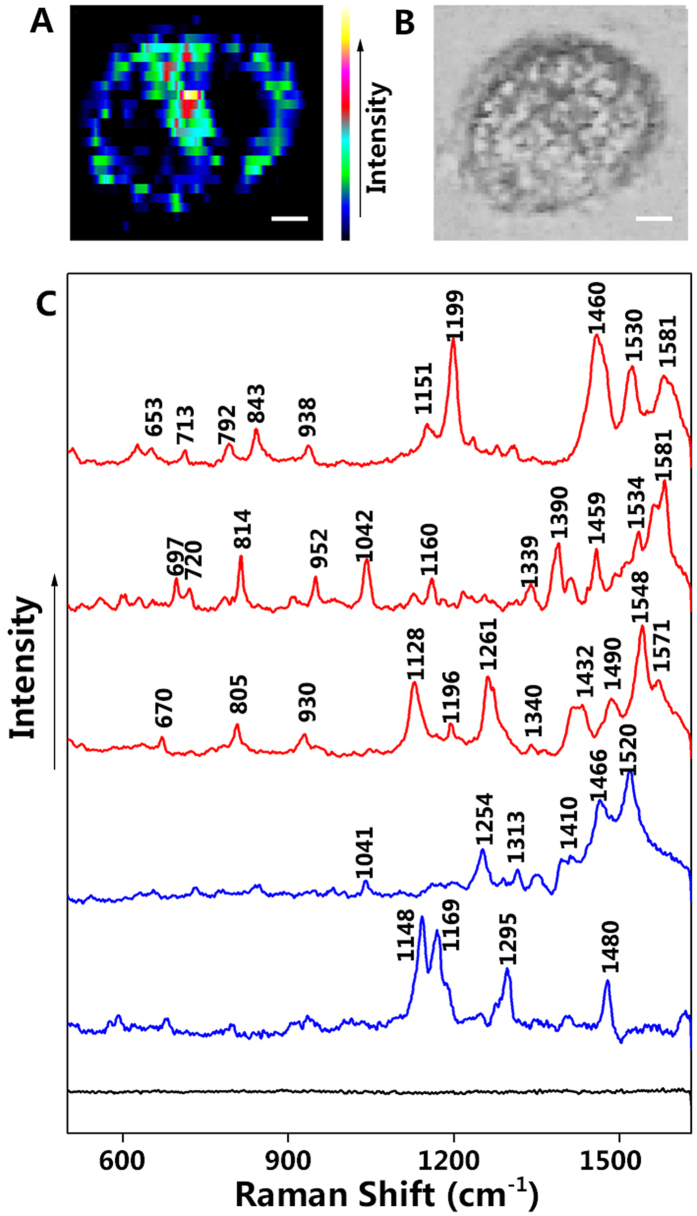
Multi-targeting SERS imaging of a HeLa cell treated with both label-free membrane- and nucleus-targeting AuNPs. (**A**) SERS image and (**B**) bright-field image of the investigated HeLa cell. (**C**) SERS spectra obtained from different positions within the cell such as membrane (blue), cell nucleus (red) and the surrounding environment (black) show significant differences in terms of their intensity and peak positions (scale bar ≡ 4 μm).

High-resolution SERS images for a living cell incubated with MBA-coated, CVa-coated, and CV-coated AuNPs are presented in panels A, B, and C of Fig. 3, respectively. MBA-coated AuNPs are designed to be distributed in the nucleus through the interactions between NLS and DNA. The Raman spectra displayed in Fig. 3A-3 are a series of representative SERS spectra of MBA dye-molecules obtained from different positions within the cell via point-by-point (1 μm × 1 μm) detection along the dotted line shown in Fig. 3A-1. The Raman intensities at 1078 cm^−1^ of these spectra were extracted and are shown in Fig. 3A-4 as histogram. When approaching the nuclear area, intense spectral response and extensive color distributions in the nucleus were observed. The blue color regions in Fig. 3A-2 indicate the presence of MBA-coated nuclear-targeting AuNPs in the single cell overlaid with a bright field image. To obtain the single cell image in Fig. 3, we used an excitation with 632.8 nm wavelength, 3.0 mW incident laser power and 1 s integration time per pixel (1 μm × 1 μm), which allowed us to obtain a 30 × 30 pixel (decided by cell size) single cell image within 40 min including the mechanical moving time in x and y direction.

Recent studies have revealed that FA and LHRH can be used as membrane-targeting molecules, since FA-Rs^27^ and LHRH-Rs^29^ are overexpressed in the HeLa cellular membrane. Using the Raman peak intensities at 1175 and 595 cm^−1^ yielded the distributions of CV-coated and CVa-coated AuNPs indicated by green and red color, respectively. Both matched with the position of the membrane in the HeLa cell (panels B and C of Fig. 3, respectively). For comparison, a SERS image of a living cell without incubation with AuNPs is shown in Figure S5 using the same measurement parameters. There, no specific Raman signals could be detected in the cell. An electron micrograph was taken to further support the observed targeting ability of the AuNPs. In the TEM image, the membrane- or nuclear-targeting AuNPs were found inside the nucleus and surrounding the cells, respectively (Figure S6). The localization of nuclear-targeting SERS AuNPs was further confirmed by a fluorescence image of the nucleus with DAPI (4′, 6-diamidino-2-phenylindole) in the same cell (Figure S7).

In most physiological processes, a series of molecular events takes place in different organelles. Therefore, the study of a single type of organelle is not sufficient for gaining a deeper-going understanding of the physiological processes. Due to the small bandwidth of the Raman peak, which yields specific chemical information of typically less than 2 nm, Raman spectroscopy yields more specific molecular information and therefore is a valuable tool in the field of multiplex detection and imaging. In this connection, the simultaneous accurate positioning of a variety of specific SERS probes in the same cell is of great importance for accessing chemical information of different cellular regions. The high lateral and axial spatial resolution in confocal Raman spectroscopy and the development of highly sensitive substrates for SERS enhancement make multi-targeting SERS imaging a very interesting technique in bioanalysis.

After having verified the localization of the NLS, FA, and LHRH-AuNPs, we now have been interested in positioning the label-free nuclear- and membrane-targeting AuNPs in the same cell to simultaneously gain biomolecular information (Raman spectra) from its membrane and nucleus (Fig. 4). AuNPs can provide strongly enhanced spectroscopic signals from the native chemical constituents of a cell due to the locally enhanced optical fields at AuNPs surfaces. There are abundant fatty acids, proteins, and nucleic acids at membrane and nucleus, resulting in very strong intrinsic Raman signals from both cellular regions (nucleus and membrane, Fig. 4A) where the label-free AuNPs were localized; representative SERS spectra from such regions are shown in Fig. 4C. Spectra from different cellular regions show clear differences both in Raman peak positions and intensities. Membrane spectra (blue) reveal peaks assigned to proteins and fatty acids, whereas spectra from the nuclear region (red) mostly show peaks characteristic of nucleic acids and proteins. For example, this can be seen in the spectral range of 500–900 cm^−1^, where significant peaks can be observed within the nuclear region of the cell (red lines, Fig. 4C) compared to fewer peaks in the membrane spectra (blue line, Fig. 4C)^30^. The detailed SERS band assignments for these spectra are given in Table 1 in the supporting information.

**Figure 5:**
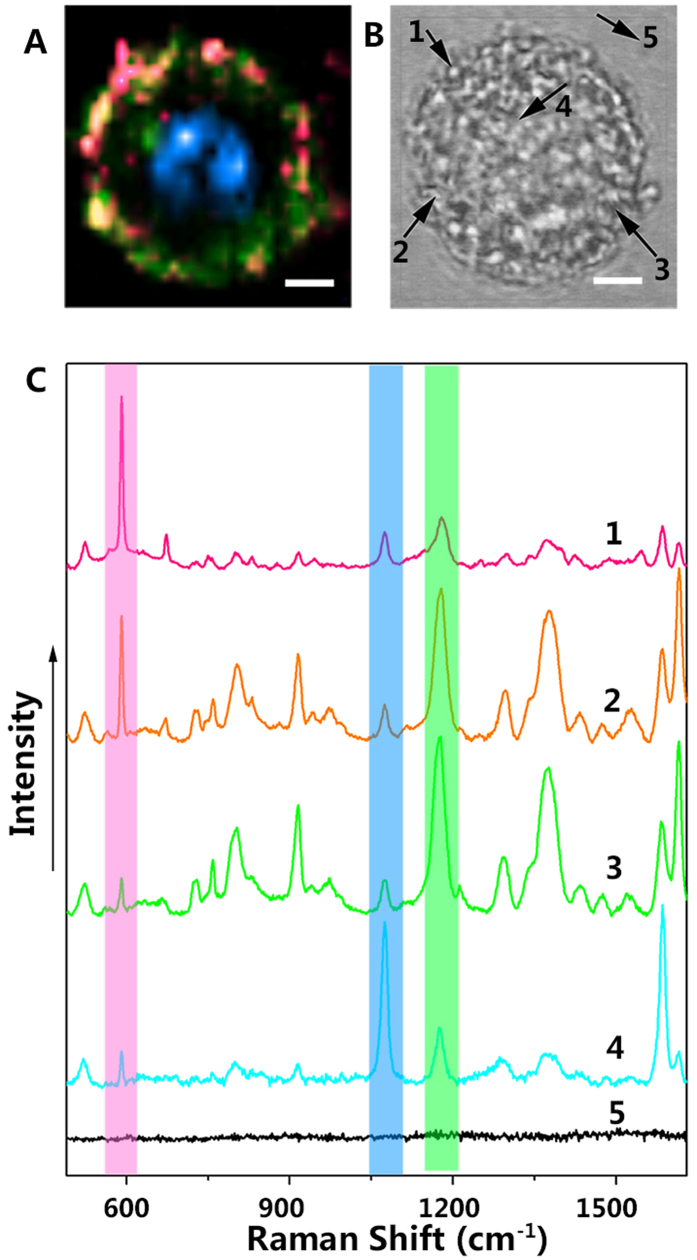
Multi-targeting SERS imaging of a HeLa cell treated with CVa-coated, CV-coated, and MBA-coated AuNPs. (**A**) Overlap of SERS images of CVa-coated AuNPs (red), CV-coated AuNPs (green), and MBA-coated AuNPs (blue). (**B**) The bright-field image of the investigated HeLa cell. (**C**) SERS spectra obtained from different positions in the cell (marked in panel B by arrows). The Raman intensities at 595, 1078, and 1175 cm^−1^ revealed the relative amount of CVa-coated, MBA-coated and CV-coated AuNPs at the corresponding positions, respectively (scale bar ≡ 4 μm).

Figure 5A shows the result of multi-targeting SERS imaging using CV-coated, CVa-coated, and MBA-coated AuNPs in the same single cell. The depth of each color reflects the signal intensity of the corresponding SERS lines. The positions of the nucleus and membrane can be clearly distinguished and are shown in different colors; the green and red region results from the SERS signals of CV and CVa depicting the outline of the cell membrane, the nuclear region can be identified from the SERS signals of MBA color-coded in blue, and the position of the yellowish color indicates the overlapping area of green and red. The SERS spectra in Fig. 5C were obtained from different positions in the cell (as indicated by black arrows in panel B). The colors used for the SERS image in panel A correspond to the colors used in panel C. The Raman intensities at 595, 1078, and 1175 cm^−1^ are reflecting the relative amount of CVa-coated, MBA-coated and CV-coated AuNPs at the corresponding position, respectively. Through the multi-targeting SERS imaging of various dye-AuNPs in the same cell, we can simultaneously track the transfer and monitor the content change of various biological macromolecules, which specifically bind to the targeting-molecules on the AuNPs.

Raman spectroscopy has played an important role not only for the investigation of physiological states (structure, composition, etc.) but also for the dynamic monitoring of physiological processes^31^. A very important bioprocess is the apoptosis (or programmed cell death), which is closely associated with a variety of complex physiological processes and diseases and therefore has attracted considerable attention^32^,^33^. A great deal of work has been done to monitor the molecular events and morphological changes in the nucleus by gel electrophoresis, flow cytometry, and terminal deoxynucleotidyl transferase-mediated dUTP nick end-labeling (TUNEL) assay, etc^34^,^35^,^36^,^37^. For this complex physiological process, SERS imaging can provide a highly comprehensive description of the physiological activities when our approach is applied where information from different cellular regions is obtained using specific targeting-AuNPs as both label-free and labelled probes.

In the following, we report our experiments where we have employed CV-coated, CVa-coated, and NLS-AuNPs to detect the apoptotic cells and obtain high-resolution SERS images. Commercially available Triton X-100 was used to induce the apoptosis process of HeLa cells^38^,^39^,^40^. In order to avoid the cell to slide off the cover slips in a long-term apoptosis process, low-concentration (30 μM) Triton X-100 aqueous solution was used in our experiment. In order to prove the effectiveness of Triton X-100 for the induction of apoptosis and the biocompatibility of the AuNPs, the viability of cells was tested by flow cytometry. The results demonstrated that the viability of cells with nuclear-targeting AuNPs and Triton X-100 incubated was reduced to 85% and 30% for up to 12 hours, respectively; for comparison, the viability of the cells with nothing was 91% (Figure S8).

**Figure 6:**
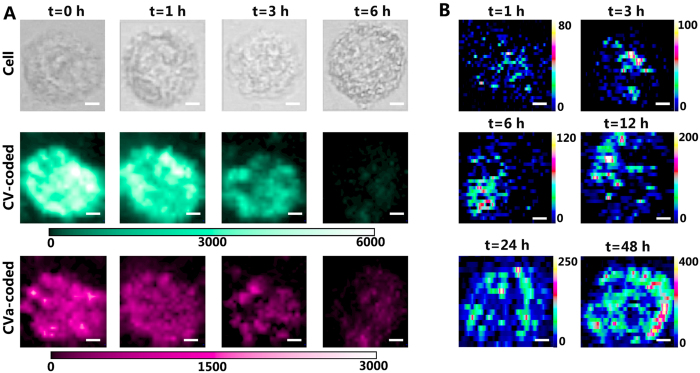
(**A**) Time-dependent SERS images (30 × 30 pixel) of HeLa cells incubated with CV- and CVa-coated AuNPs after 0, 1, 3, and 6 h of interaction with 30 μM Triton X-100. (**B**) Time-dependent SERS images of HeLa cells with added label-free nucleus-targeting AuNPs, after 1, 3, 6, 2, 24, and 48 h of interaction with 30 μM Triton X-100. A laser power of 3.0 mW was chosen and each pixel was exposed for 1 s (scale bar ≡ 4 μm).

To study the detachment of the membrane-specific proteins (FRs and LHRH-Rs) during apoptosis, CV-coated and CVa-coated membrane-targeting AuNPs were incubated with the same cells for 2 h before Triton X-100 was added. High-resolution SERS images of HeLa cells treated with apoptosis inducer for different times (here, 0, 1, 3, and 6 h) are presented in Fig. 6A. Notably, the HeLa cells produced strong SERS signals at 0 h of incubation, which are significantly reduced at 1 and 3 h. With prolonging the incubation time up to 6 h, the SERS signals dramatically dropped nearly below the detection limit. Raman images of cells with longer incubation time are not shown, since no signal could be detected anymore. Dye-coated AuNPs were linked to FRs and LHRH-Rs for the FA and LHRH bridging; thus, the SERS intensities of CV and CVa clearly reflect the relative concentration of FRs and LHRH-Rs on the membrane. Triton X-100 results in a detachment of membrane protein from the membrane surface. As a result, LRs and LHRH-Rs continuously reduced with incubation time after apoptosis inducer treatment and the SERS intensity of CV and CVa correspondingly decreased at different apoptotic stages. The result could be further confirmed by fluorescence images of HeLa cells, which were incubated with QDs conjugated FA, comparing cells without and with Triton X-100 treatment for 12 h (Figure S9). Different from our previous confocal settings, here, the pinhole was adjusted to 200 μm to increase the detection region in the z-direction, allowing for the detection of signals of Raman reporters from the whole cellular membrane.

Figure 6B shows that the intrinsic SERS signals of the DNA in the cell are sensitively responsive at different stages of apoptosis; the corresponding bright-field images are shown in Figure S10. The high-resolution SERS imaging of HeLa treated with apoptosis inducer for different times (1, 3, 6, 12, 24, and 48 h) was constructed from the total SERS intensity between 490 and 1630 cm^−1^. Specifically, a weak Raman signal (the intensity on the scale used is ~80) and a relatively uniform distribution of DNA are observed in HeLa cells after 1 h incubation. However, at the early apoptosis stage after 3 h of incubation, the SERS signals increased to a higher level (~100) due to increased DNA concentrations resulting from diminishing nuclei and slight condensation of chromatin, which is observed from the distribution in the SERS mapping image. The chromatin further condensed at 6 h and 12 h, and, consequently, the SERS signals further increased to higher levels (~120 and ~200, respectively). After 24 h of incubation, the nuclei have been severely damaged; the main change is due to chromatin decomposition and nuclear membrane rupture. The nucleoplasm contains a large number of DNA-histone complexes, which are formed by the interaction between positively charged alkaline amine acids (mostly arginine and lysine) and negatively charged phosphate groups in double-stranded DNA. Therefore, a large number of Raman-active molecules leak from the nucleus, such as DNA fragments, arginine, lysine, and so on. In the process of cell metabolism, a considerable number of AuNPs failed to enter the nucleus, which now can enhance the Raman signals of the molecules escaping the cells due to leakage. Therefore, the higher level SERS signals of ~250 and ~400 are distributed over all regions of the cell after 24 and 48 h of incubation, respectively. When the incubation time increases continually, the cells started falling from the cover slips in a long-term apoptosis process. Therefore, no more reasonable SERS images could be acquired for longer incubation times.

From the SERS results presented above, we learn that apoptosis is a slow process lasting for about 48 h and entailing a variety of biochemical events, such as protein denaturation, protein degradation, DNA fragmentation, and macromolecular synthesis^41^,^42^. During the progress of cell apoptosis, the physiological processes become more complicated, since the number of molecular events increases gradually. In order to simplify the problem, the 150–200 characteristic spectra from numerous cells at each incubation time were chosen for data analysis and classified into 6 categories according to the following scheme: the spectra after 1 h of incubation represent category “a”; after an incubation time of 3 h some of the spectra taken have changed in comparison to the category “a” spectra; they form the new category “b”. Spectra different from categories “a” and “b” after an incubation time of 6 h are classified as category “c” and so on. The six temporal fix points used for the categorization are 1, 3, 6, 12, 24, and 48 hours. Since “hot-spot” effects in SERS spectroscopy result in singular spectral intensity variations, the peak intensities are not considered for the analysis procedure.

Clustering techniques have become an important tool for the analysis of data obtained from spectroscopy. Here, data analysis was carried out with a multivariate, unsupervised data clustering technique of the K-means method using MATLAB R2014a employing a graphical user interface toolkit. More precisely, every spectrum is represented as its sparse coding to reduce the number of dimensions. Taking the sparse coding of spectra as the input, 370 clusters are received with K-means. According to the first time of appearance of each cluster, the 370 clusters are organized by 6 categories (see above). The classification result is shown in Figure S11, where similar spectra belonging to the same categories are represented by the same color. It should be pointed out, that the spectral peaks, the essence part of sparse coding, are used to show the classification performance. The spectral differences of the 6 categories are shown in Figure S12, considering the spectral peaks at 819 and 1509 cm^−1^. It will become clearer when more peaks are considered.

We can learn what specific molecular events happened during apoptosis by focusing on the spectral differences of the 6 categories. The differences for the 6 categories of spectra are shown in Figure S13. The Y-axis of the six curves displayed there gives the probability (percentage) for observing Raman peaks at the corresponding wavenumber positions. This percentage is proportional to the number of molecules exposed to the plasmonic fields of the NT-AuNPs. From this, the change of molecular concentration can be determined by analyzing the intensity differences for all 6 categories. The percentage values at 1500 and 1530 cm^−1^, which are the characteristic peaks of DNA, increased noticeably after 3 to 6 h of incubation with the apoptosis inducer, pointing to the increased DNA concentrations resulting from the diminishing nuclei and the chromatin condensation at the early apoptosis stage. The percentage values decline after 12 to 48 h, which can be attributed to DNA fragmentation^43^,^44^,^45^. The increase of the percentage values of the protein bands at 1178, 1230, and 1275 cm^−1^ after 6 h of incubation reflects the generation of biomacromolecules (proteins) and the subsequent decrease shows the protein denaturation and degradation.

By incubation with CV-coated and CVa-coated membrane-targeting AuNPs and label-free nuclear-targeting AuNPs, we have successfully monitored the processes of biomolecules transformation, such as membrane protein dissociation, chromatin condensation, and DNA fragmentation during cellular apoptosis after the treatment with 30 μM Triton X-100. Based on the time-dependent results, we can roughly describe the cell apoptosis process considering the membrane proteins and nucleic acid.

At the beginning, after the apoptosis process of HeLa cells induced by Triton X-100, a series of physiological changes follows: membrane proteins (FRs and LHRH-Rs) are gradually detached, nuclei diminish, and chromatin slightly condenses, which progresses during the next 3 hours. When the incubation time is increased to 6 hours, most of the membrane proteins have fallen off. After 24 h of incubation, the nuclear membrane is ruptured, which indicates the arrival of the end stage of apoptosis. From that point of time on, the cells begin to die and fall off from the cover slips, gradually.

**Figure 7:**
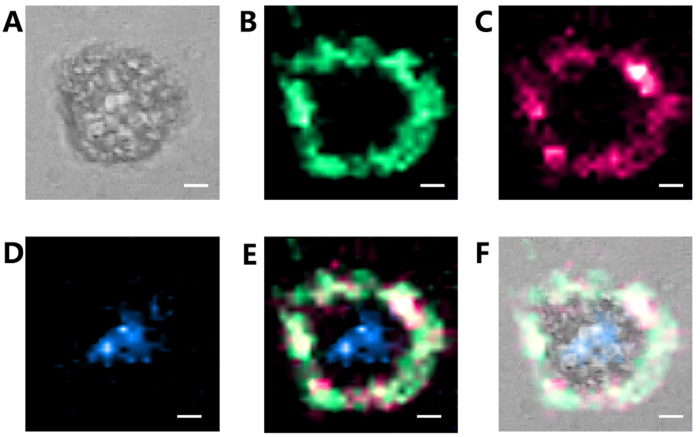
High-resolution SERS images (30 × 30 pixel) of the same HeLa cell treated with CV-coated, CVa-coated, and label-free nuclear-targeting AuNPs. (**A**) Bright-field image and SERS images of (**B**) CV-coated AuNPs, (**C**) CVa-coated AuNPs, and (**D**) label-free nucleus-targeting AuNPs. (**E**) Overlapping image of (**B–D**) (scale bar ≡ 5 μm).

According to the results shown in Figs 4 and 5, we believe that multi-targeting SERS imaging using various dedicated SERS probes in the same cell is a powerful tool for gaining a deeper understanding of physiological processes. Figure 7 shows the result of multi-targeting SERS imaging using labelled CV-coated and CVa-coated membrane-targeting AuNPs, and label-free nuclear-targeting AuNPs in the same single cell, which allows to obtain intrinsic Raman signals from biochemicals of the nucleus and trace the distribution of membrane proteins (FA-Rs and LHRH-Rs) in the same cell.

In conclusion, we have successfully presented a simple and universal synthesis method of SERS probes for label and label-free nuclear and membrane-targeting SERS imaging. Using these probes, we have obtained simultaneously three-dimensionally spatially resolved label and label-free images of nucleus and membrane in the same single HeLa cells by utilizing surface-enhanced Raman spectroscopy combined with a confocal microscopy arrangement. Labelled membrane targeting (CV-coated and CVa-coated AuNPs) and label-free nuclear targeting were employed for the investigation of cells during apoptosis after treatment with 30 μM Triton X-100. We found that membrane protein dissociation and chromatin condensation take place at early stages of apoptosis for approx. 6 h and DNA fragmentation at the later stage of apoptosis, after approx. 12 h of incubation. In most physiological processes, the molecular events of various organelles influence each other within the cell. Therefore, the study of a single organelle’s change separately is not sufficient for obtaining a more detailed insight into physiological processes. The proposed SERS sensing strategy, which allows for multi-targeting and three-dimensional SERS imaging of single cells, provides an attractive 2D/3D tracing method on subcellular level, which is of great significance for the detection of complex physiological processes

## Methods

### Preparation of organelle-targeting dye-coated (labelled) AuNPs

Citrate-stabilized gold nanoparticles (AuNPs) with an average diameter of ~40 nm for SERS studies were synthesized by reduction of gold ions (HAuCl_4_) using trisodium citrate. In a typical procedure, 1.5 mL of 1% trisodium citrate was added to the 100 mL solution of 0.01% HAuCl_4_ after the gold solution was heated to 120 °C. Heating and stirring were continued for 20 min after the color change from colorless to red was observed, and the solution was allowed to reach room temperature. Finally, the nanoparticles were re-dispersed using DI water as dispersant by ultrasonication after centrifugation at 4000 rpm for 20 min. Transmission electron microscopy (TEM) indicated that the nanoparticles had an average diameter of 40 ± 5 nm, and UV-VIS spectroscopy showed a surface plasmon resonance band at 532 nm.

After incubated with a small amount of 1 mM ethanol solution of dye-molecules (CV (5 μL), CVa (10 μL), or MBA (20 μL)), poly allylamine (PAH) (MW = 17000) was applied to the particles‘ surfaces to reduce aggregation and supply –NH^2^. After the dye-particles were diluted in DI water to a concentration of ~10^12^ particles/mL, the nanoparticles were subsequently modified with PAH by adding a 1 mL aliquot of a 0.1% (w/w) aqueous solution of PAH to 10 mL of the aqueous nanoparticle suspension. The PAH-AuNPs were allowed to incubate overnight at room temperature under gentle shaking.

After successful modification with PAH, the AuNPs were conjugated with the targeted molecule utilizing the EDC-NHS reaction. A small amount of targeted molecule solution (NLS (20 μL, 1 mg/mL), FA (200 μL, 0.1 mM), or LHRH (20 μL, 1 mg/mL)) was mixed with 100 μL of 1 mM 1-ethyl-3-[3-dimethylaminopropyl] carbodiimide hydrochloride (EDC) and 40 μL of 1 mM N-hydroxysuccinimide (NHS). The mixture was then incubated at room temperature for 45 min and afterwards added to 10 mL of the modified nanoparticle solution. Gentle stirring was continued for 24 h at room temperature. Finally, the product was centrifuged and re-dispersed in DI water to a final concentration of 10^10^–10^11^ particles/mL. The success of peptide and PAH conjugation was confirmed through UV-VIS spectroscopy and TEM.

### Preparation of the organelle-targeting label-free AuNPs

The synthesis procedures for organelle-targeted label-free AuNPs and organelle-targeted dye-coated AuNPs were similar. However now, the conjugation of dye-molecules was unessential before PAH modification.

### Cell culture and targeting

Human cervix carcinoma cell line (HeLa) was purchased from the China Center for Typical Culture Collection (CCTCC) and cultured in Dulbecco’s modified Eagle medium (DMEM) supplemented with 10% heat-inactivated newborn calf serum, 1% (w/v) glutamine, and 1% penicillin-streptomycin under a humidified atmosphere (5% CO_2_ plus 95% air) at 37 °C.

(a) HeLa cells were seeded onto an 18 mm cover slip placed in a 35 mm Petri dish for 12 h in 3 mL supplemented DMEM culture medium after re-suspension using fresh 0.25% trypsin, 1 mM EDTA-Na.

(b) 150 μL of the nuclear-targeting AuNPs were added, while shaking the dish gently to ensure good mixing. The dish was then incubated in a humidified 5% CO_2_ atmosphere for 24 h at 37 °C. Afterwards, the cover slips were rinsed three times with PBS and the 3 mL fresh culture medium was supplemented.

(c) 150 μL of the membrane-targeting AuNPs were added, while shaking the dish gently to ensure good mixing. Like for the nuclear-targeting AuNPs, the dish was then incubated in a humidified 5% CO_2_ atmosphere for 24 h at 37 °C. Afterwards, the cover slips were rinsed three times with PBS and the 3 mL fresh culture medium was supplemented.

Note: According to the experimental purpose, steps (b) and (c) and SERS AuNPs were selectively employed.

### Cellular uptake of membrane-targeting AuNPs during Triton X-100-induced apoptosis

The HeLa cells on the cover slip were treated with Triton X-100 (30 μM) for 0, 1, 3, and 6 h at 37 °C in a 5% CO_2_ incubator. After the cells were washed three times with PBS, a culture medium containing two kinds of membrane-targeting AuNPs were added and incubated for 2 h. Before measurement, the cells were rinsed three times with PBS.

### Internalization of nuclear-targeting AuNPs during Triton X-100-induced apoptosis

The HeLa cells were seeded onto the cover slip and incubated for 12 h. The culture medium was then replaced by a culture medium containing nuclear-targeting AuNPs (150 μL) and incubated for 24 h at 37 °C. The cells on the cover slip were treated with Triton X-100 (30 μM) for 0, 3, 6, 12, 24, and 48 h at 37 °C in a 5% CO_2_ incubator and then rinsed three times with PBS.

### SERS Imaging

The sample used for the SERS imaging was prepared by reversely placing the cover slip on a fluted glass slide filled with PBS. SERS spectra were acquired using a Jobin Yvon Raman micro spectrometer (HR800), which utilized a 632.8 nm excitation laser (He-Ne laser) and a microscope with a 50× objective lens. Raman spectra were collected once with 1 s integration time over a spectral range from 490 to 1630 cm^−1^. When SERS imaging was performed, the scan over a single living cell was carried out on a computer-controlled XY stage in 1 μm steps at a laser spot size of ~1 μm^2^. SERS images of the label-free AuNPs were generated using the integrated signal-to-baseline intensity from 490 to 1630 cm^−1^, and SERS images of the dye-coated AuNPs were generated using the intensity of the 595 cm^−1^ (CVa), 1175 cm^−1^ (CV), and 1078 cm^−1^ (MBA) Raman lines.

### Calculation and K-means Cluster Analysis

Data analysis was carried out using MATLAB R2014a employing a graphical user interface toolkit. Firstly, the dimension of the data set had to be reduced. Each original Raman spectrum has 1016 dimensions. We have simplified each Raman spectrum with sparse coding using the BPFA algorithm to reduce the number of dimensions from 1016 to 300, which still can capture the original data set’s essential features. Then, we have clustered the Raman spectra into 370 clusters using their sparse coding using the k-means method. Finally, we have categorized the above 370 clusters into 6 categories. Based on the first-come-first-serve algorithm (FCFS), the 370 clusters were organized in 6 categories according to the first appearance of each cluster. The spectra after 1 h of incubation represent category “a”; after an incubation time of 3 h, some of the spectra have changed in comparison to the category “a” spectra, which represents the new category “b”. Other new categories can be generated by repeating the process above.

## Additional Information

**How to cite this article**: Chen, Y. *et al*. Combined Labelled and Label-free SERS Probes for Triplex Three-dimensional Cellular Imaging. *Sci. Rep.* **6**, 19173; doi: 10.1038/srep19173 (2016).

## Supplementary Material

Supplementary Information
