# Supplementary material for: Combined Labelled and Label-free SERS Probes for Triplex Three-dimensional Cellular Imaging
Source: Sci Rep. 2016 Jan 19;6:19173. doi: 10.1038/srep19173 (PMC4726017; doi:10.1038/srep19173)
Supplement: Supplementary Information [file srep19173-s1.doc]

*Supplementary Information*

Combined Labelled and Label-free SERS Probes for Triplex Three-dimensional Cellular Imaging

Yong Chen^1^, Xiangru Bai^1^, Le Su^1^, Zhanwei Du^2^, Aiguo Shen^1^*, Arnulf Materny^3^, Jiming Hu^1^*

^1^ Key Laboratory of Analytical Chemistry for Biology and Medicine, Ministry of Education, College of Chemistry and Molecular Sciences, Wuhan University, Wuhan 430072 (China)

E-mail: J. Hu ([jmhu@whu.edu.cn](mailto:jmhu@whu.edu.cn))

E-mail: A. Shen ([agshen@whu.edu.cn](mailto:agshen@whu.edu.cn))

^2^ College of Computer Science and Technology, Jilin University, Changchun 130012 (China)

^3^ Department of Physics & Earth Sciences, Focus Area Health, Jacobs University Bremen, Campus Ring 1, 28759 Bremen (Germany)


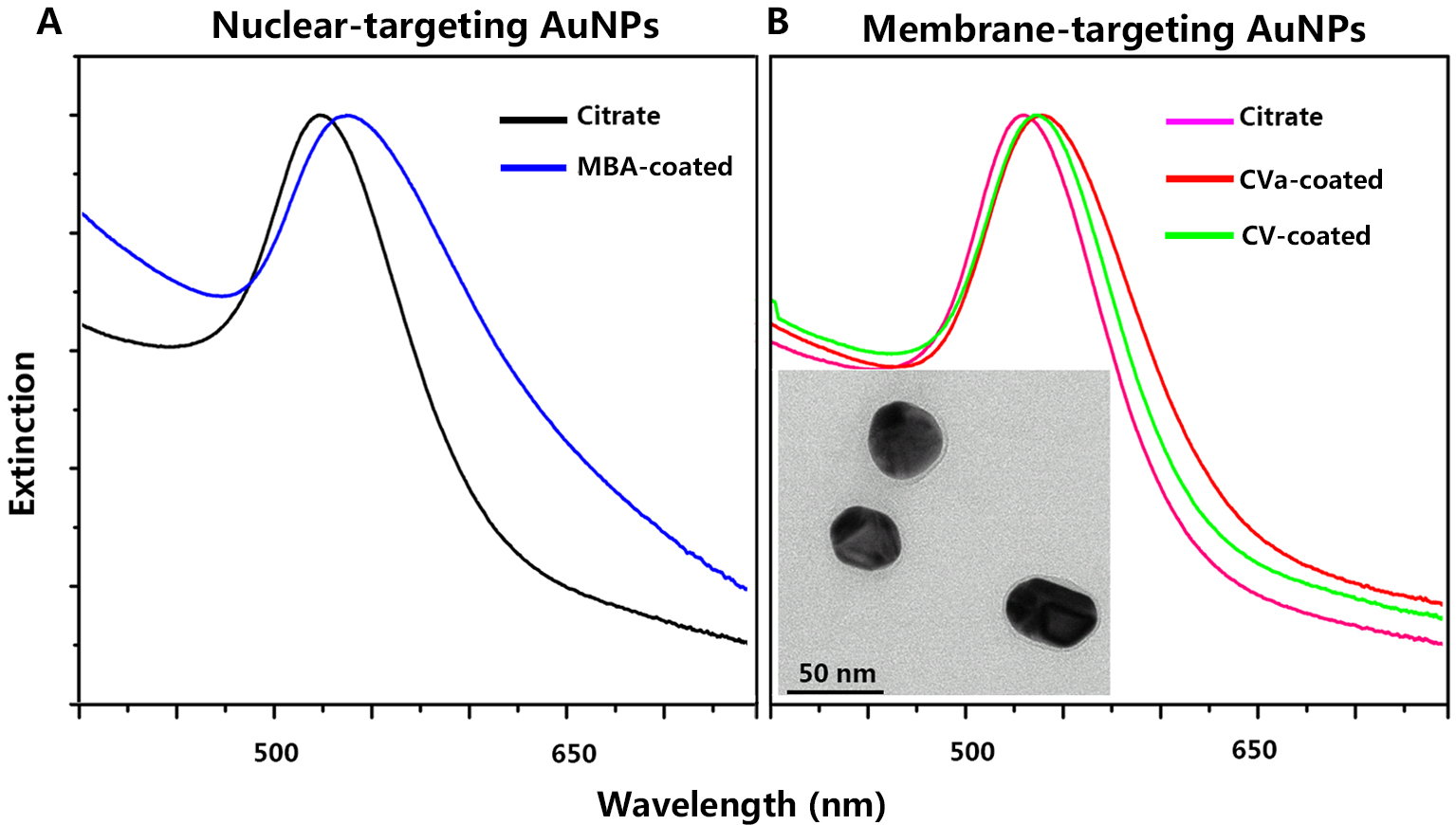


**Figure S1.** Characterization of AuNPs. UV-VIS spectra of (A) pure AuNPs (black) and nuclear-AuNPs (blue); (B) pure AuNPs (pink), CVa-coated membrane-AuNPs (red) and CV-coated membrane-AuNPs (green) in aqueous solution. The inset shows a TEM image of targeting AuNPs. There is a slight red shift in the SPR peak and an approx. 3 nm thick low-contrast layer surrounding the metal indicating successful conjugation.


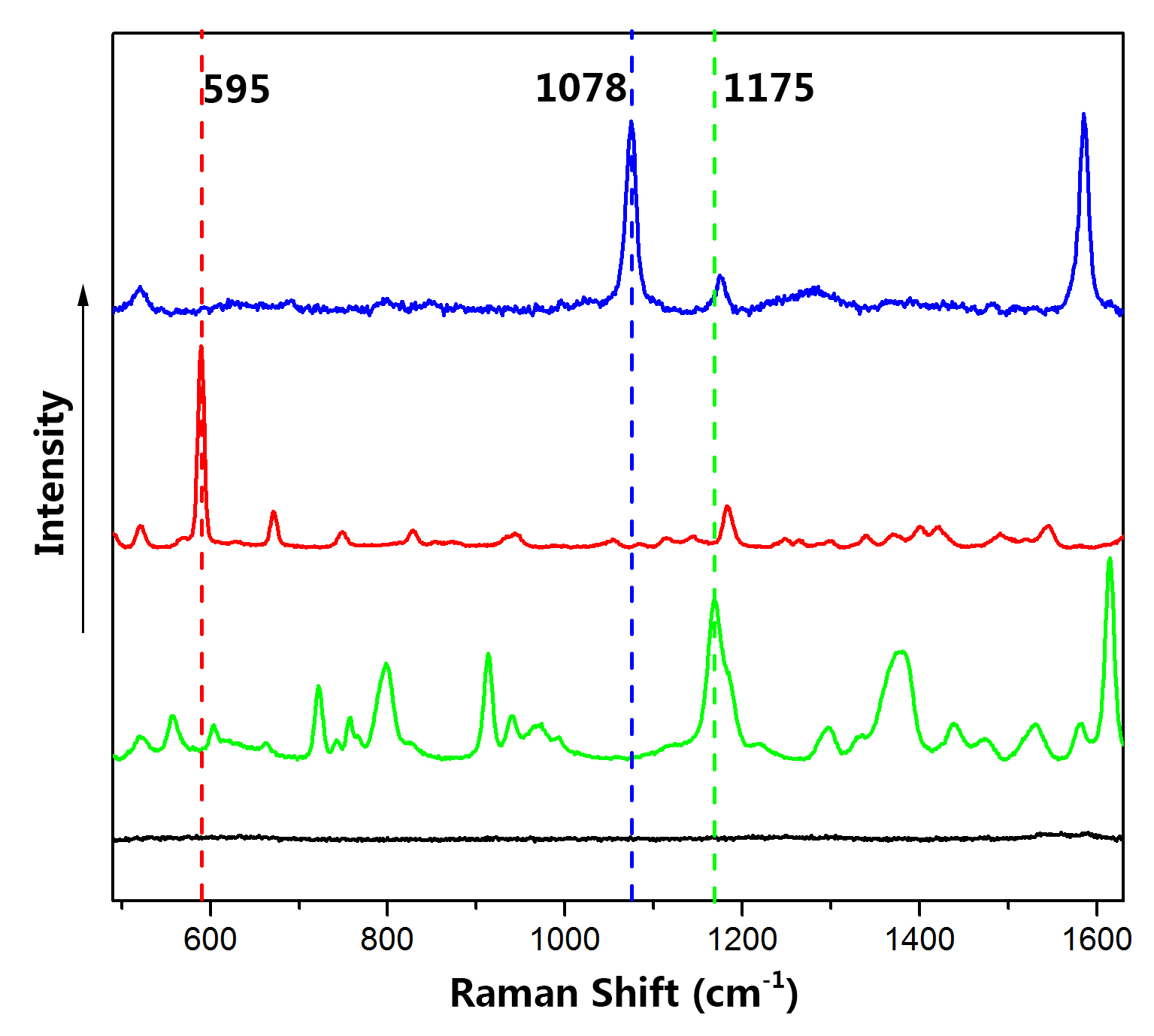


**Figure S2.** SERS spectra of MBA-coated AuNPs (blue), CVa-coated AuNPs (red), CV-coated AuNPs (green), and label-free AuNPs (black).


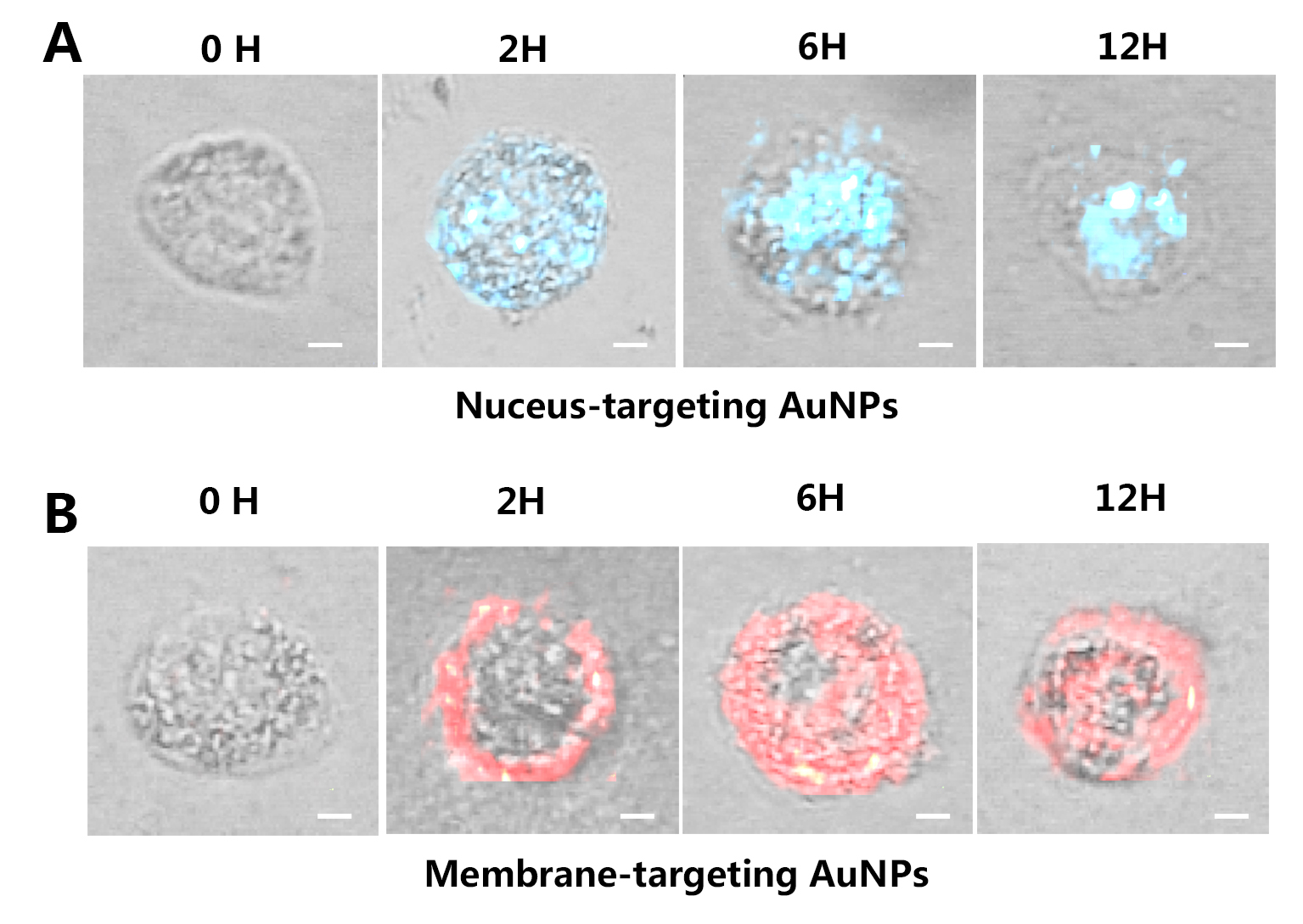


**Figure S3.** SERS images of a HeLa cell incubated with (A) MBA-coated nuclear-targeting AuNPs and (B) CV-coated membrane-targeting AuNPs for 0, 2, 6, and 12h.


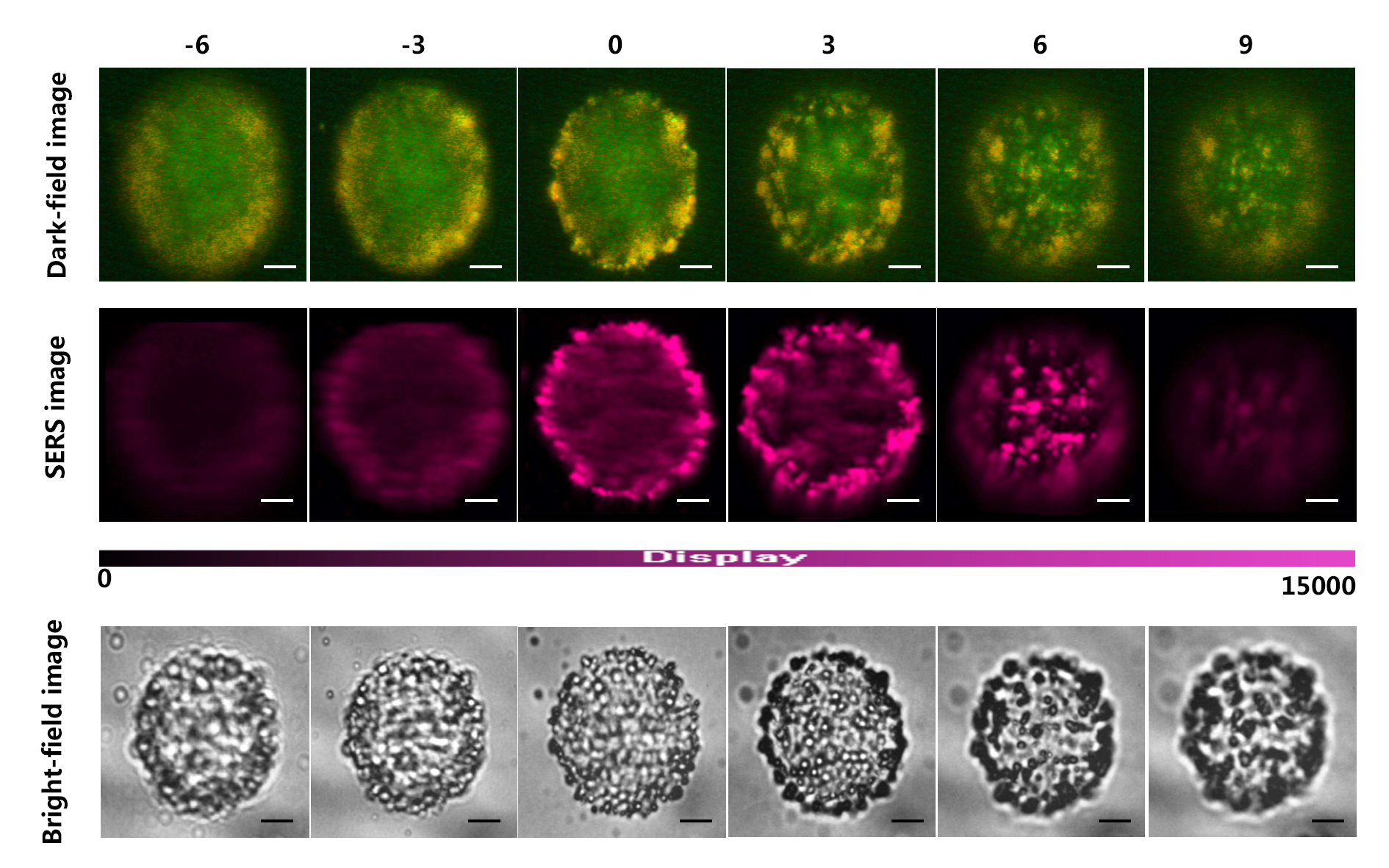


**Figure S4.** High-resolution SERS images obtained from different “z-slices” of the same cell by adjusting the focal plane in z-direction. The dark-field image and bright-field image are corresponding to the SERS images.


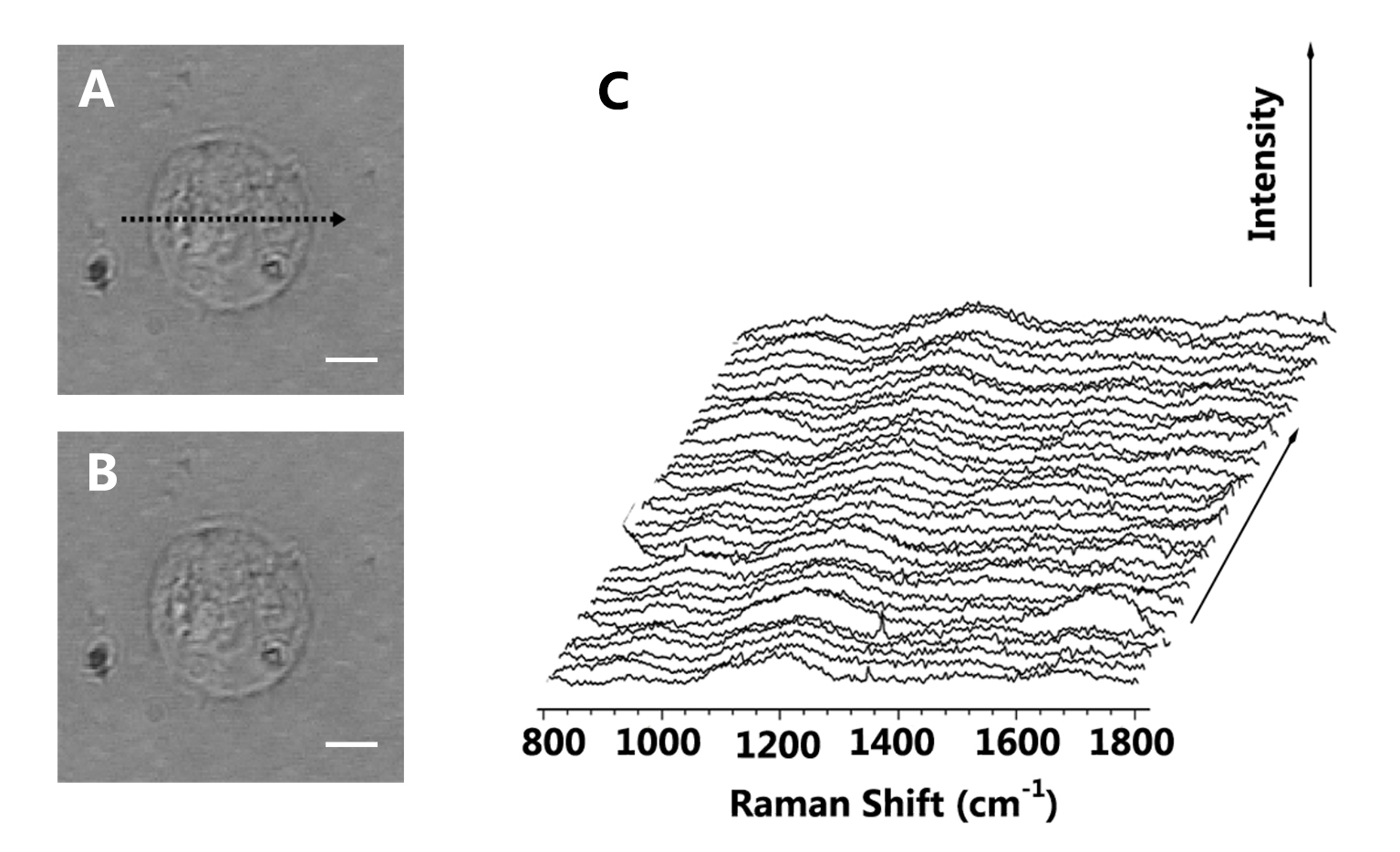


**Figure S5.** SERS images of a HeLa cell without incubation with AuNPs. (A) Bright-field image and (B) overlap image of bright-field image and SERS image. (C) SERS spectra obtained from different positions within the cell via point by point detection along the dotted line in A.

**
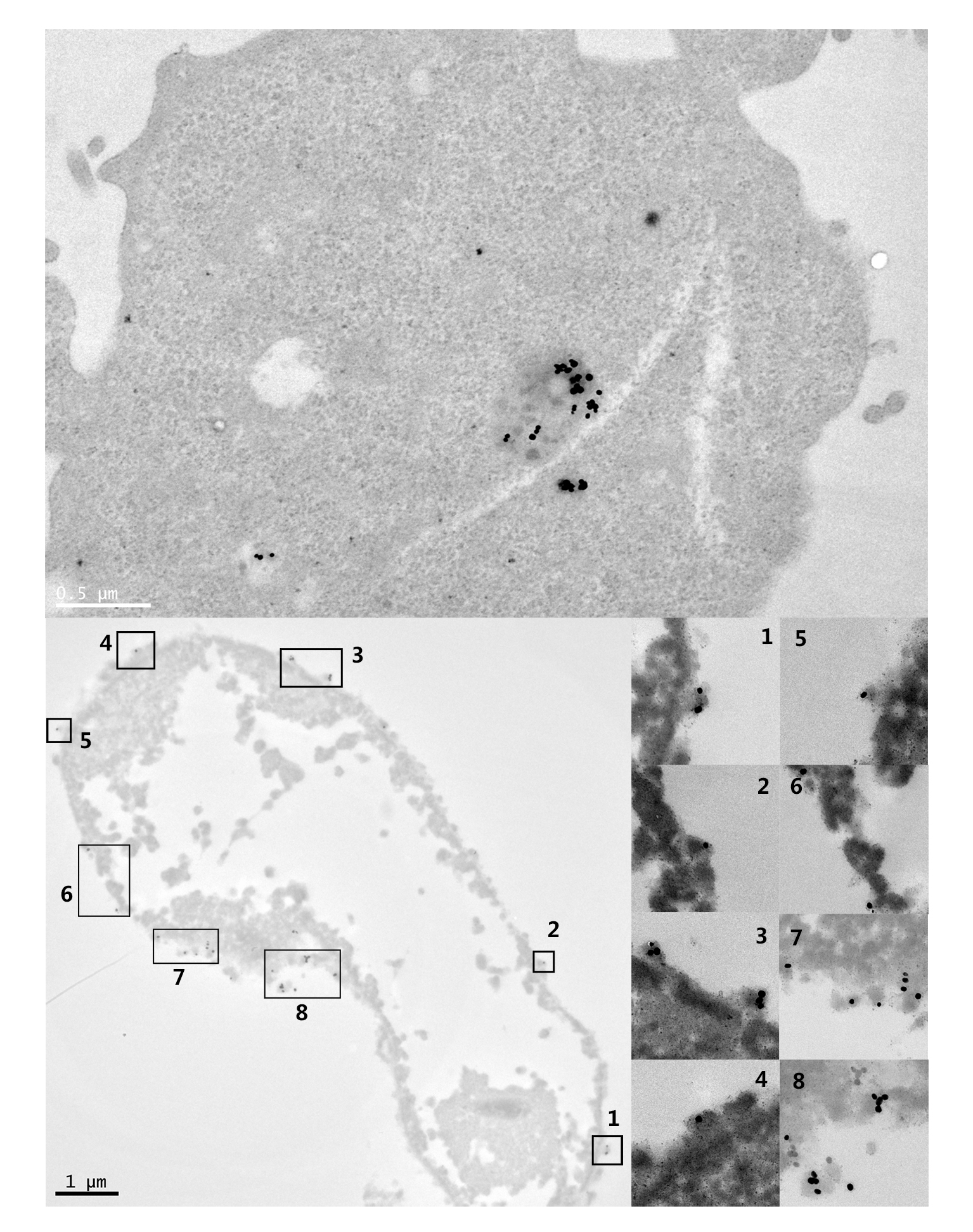
**

**Figure S6.** TEM images of (a) NT-AuNPs in the cell nucleus and (b) MT-AuNPs surrounding the HeLa cell.


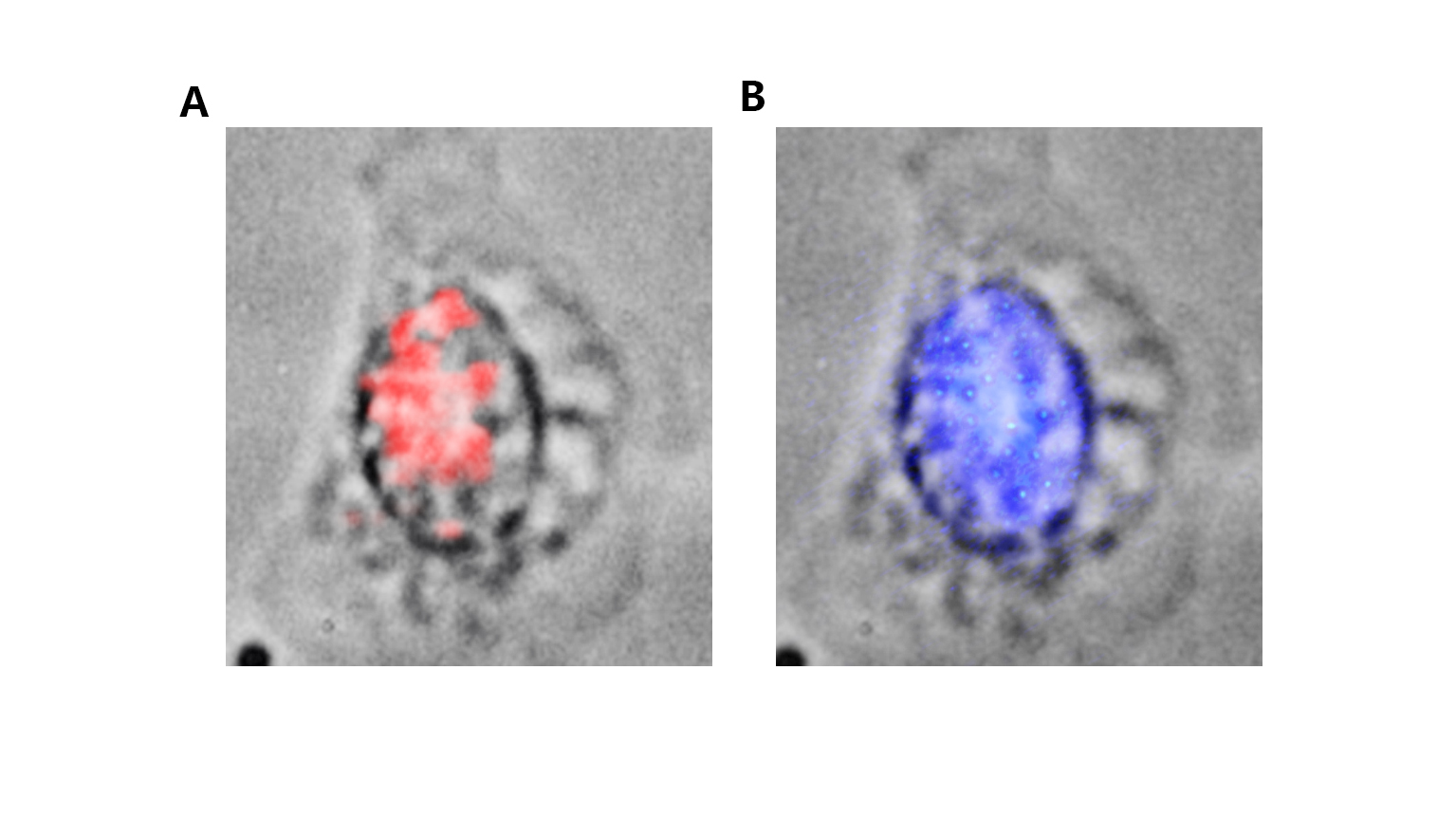


**Figure S7.** (A) SERS image of nuclear-targeting AuNPs and (B) fluorescence image of DAPI staining nucleus in the same cell.

**Table 1. Raman frequencies observed in SERS spectra of HeLa cells and their tentative assignments**

| Raman bands (cm^-1^) | Assignments |
| --- | --- |
| 642  653 | Tyr: ν(CS)  Tyr: γ(CC) |
| 670 | T, G |
| 720 | Lipid |
| 792 | C, T, DNA, O-P-O |
| 805 | O-P-O, RNA |
| 843 | Glycolipid |
| 938 | Peptide: ν(CC) |
| 952 | C-C |
| 1128 | Proteins: (C-N), carbohydrates: (C-O) |
| 1148 | Deoxyribose-phoshpate |
| 1160 | Proteins, ν(C-N), ν(C-C), ρ(CH_3_) |
| 1254 | Lipids |
| 1295 | Proteins: amide Ш, lipids |
| 1313 | Lipid |
| 1410 | ν(COO^-^) |
| 1432 | A, G |
| 1454 | Deoxyribose, δ(CH2) |
| 1480 | A, G |
| 1490 | A, G |
| 1530 | G, C |
| 1534 | A, C, G |
| 1548 | Proteins: amide П |
| 1581 | A, G |


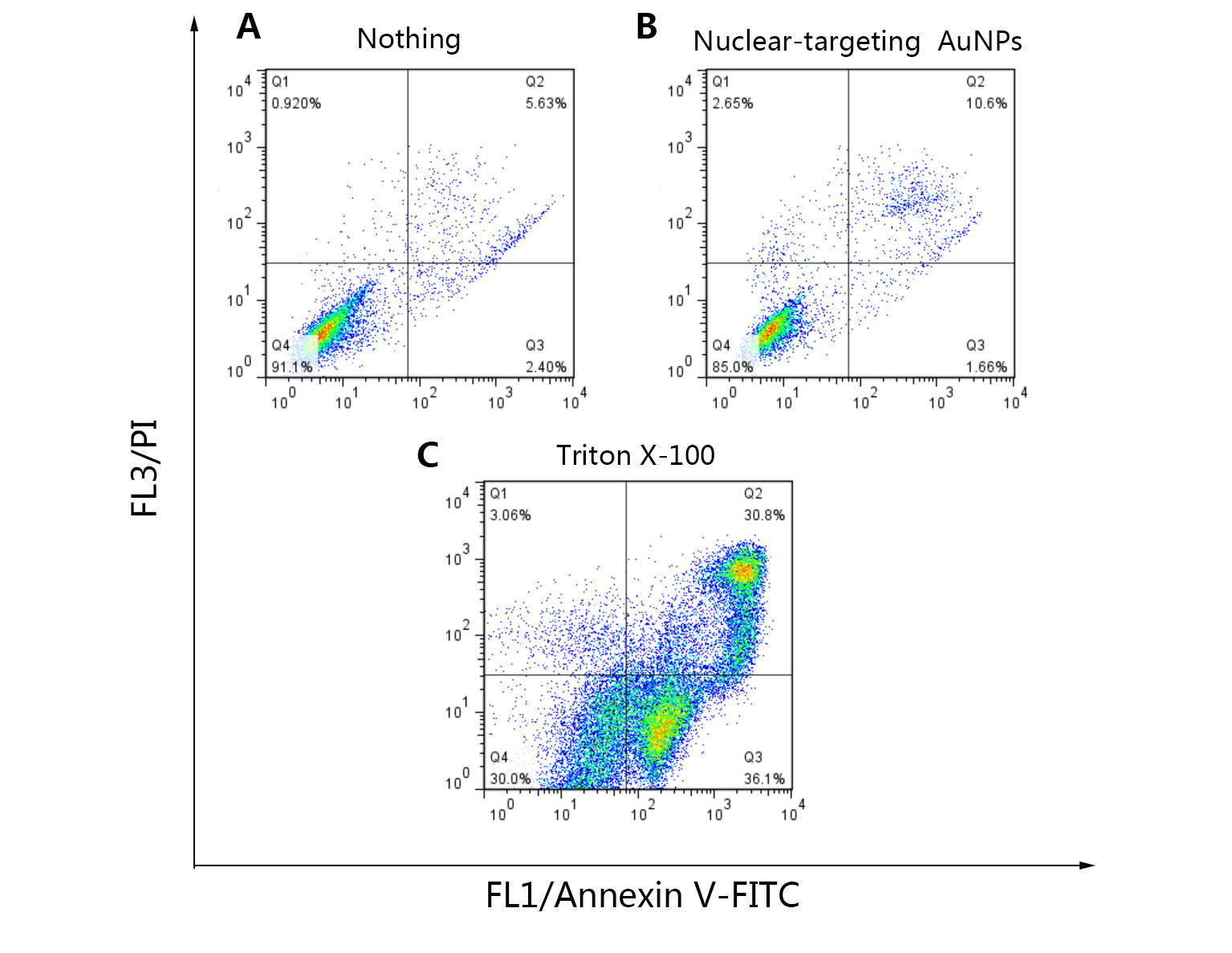


**Figure S8.** The flow cytometry analysis results of cells (A) without / (B) with nuclear-targeting AuNPs and (C) with Triton X-100 for up to 12 hours incubation time, respectively.

**
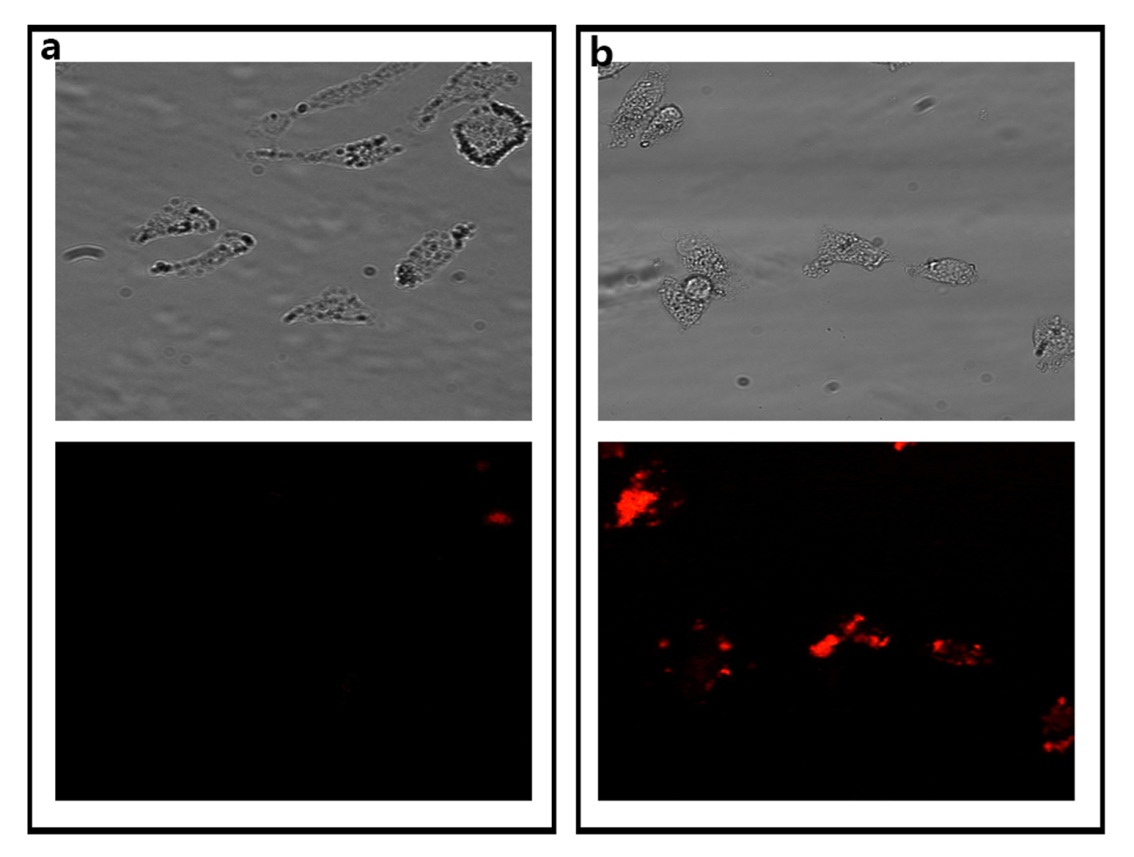
**

**Figure S9.** Fluorescence images of HeLa cells incubated with QDs conjugated to FA treated (a) with and (b) without Triton X-100 for 12h.


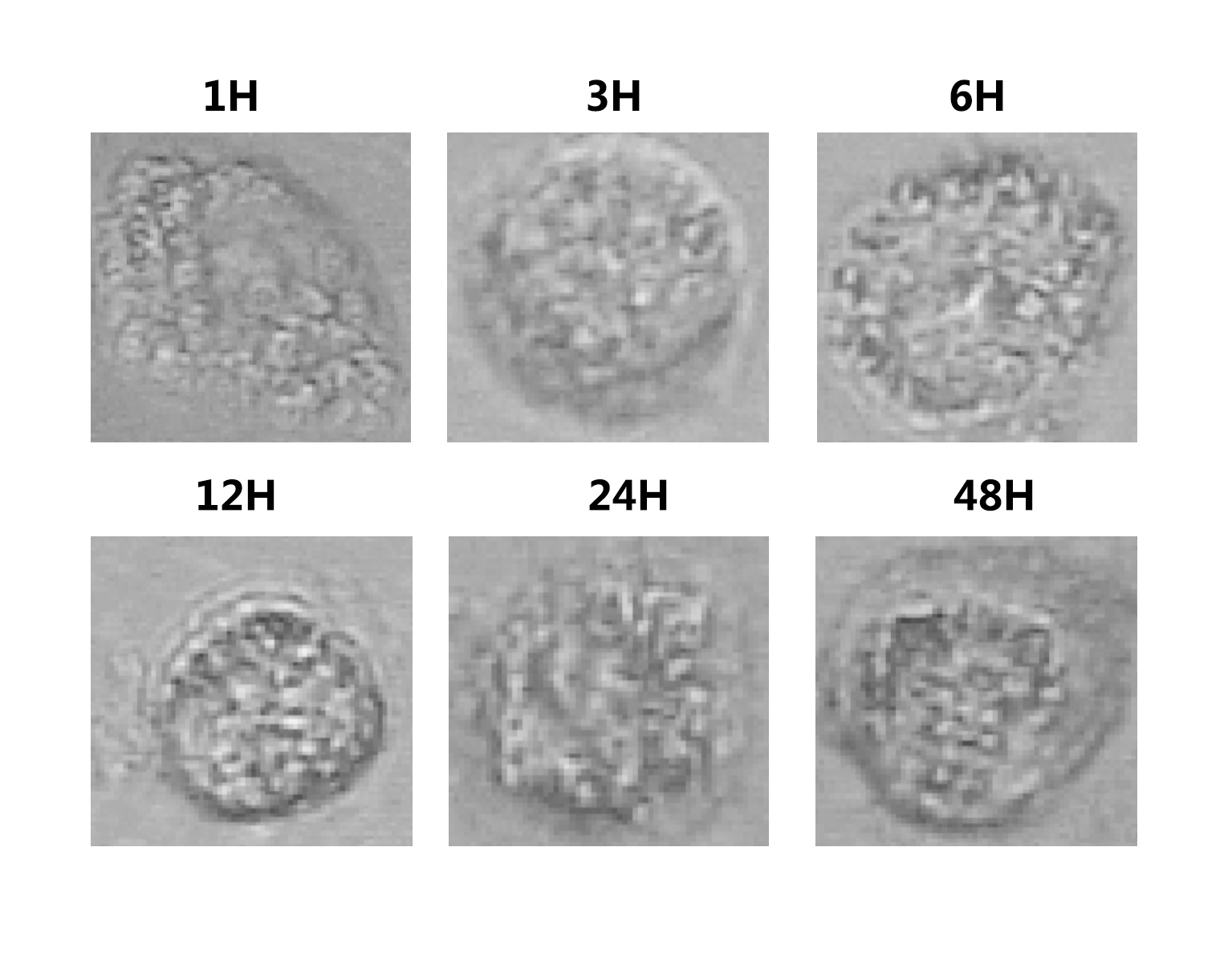


**Figure S10.** Bright-field images of a HeLa cell at different stages of apoptosis, corresponding to the SERS images shown in Figure 6B.


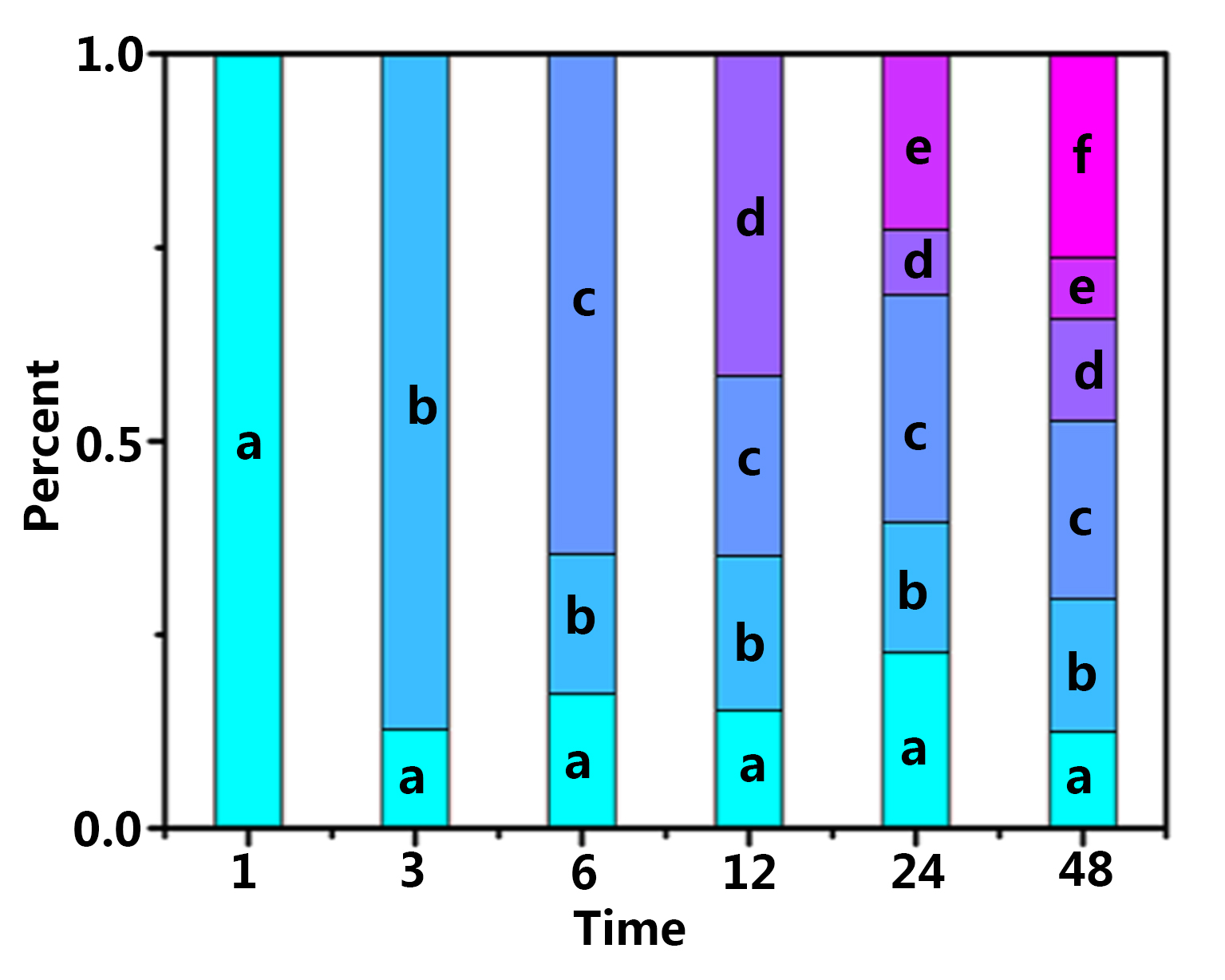


**Figure S11.** Results from a K-means (KM) clustering analysis of the apoptosis process as a function of time.


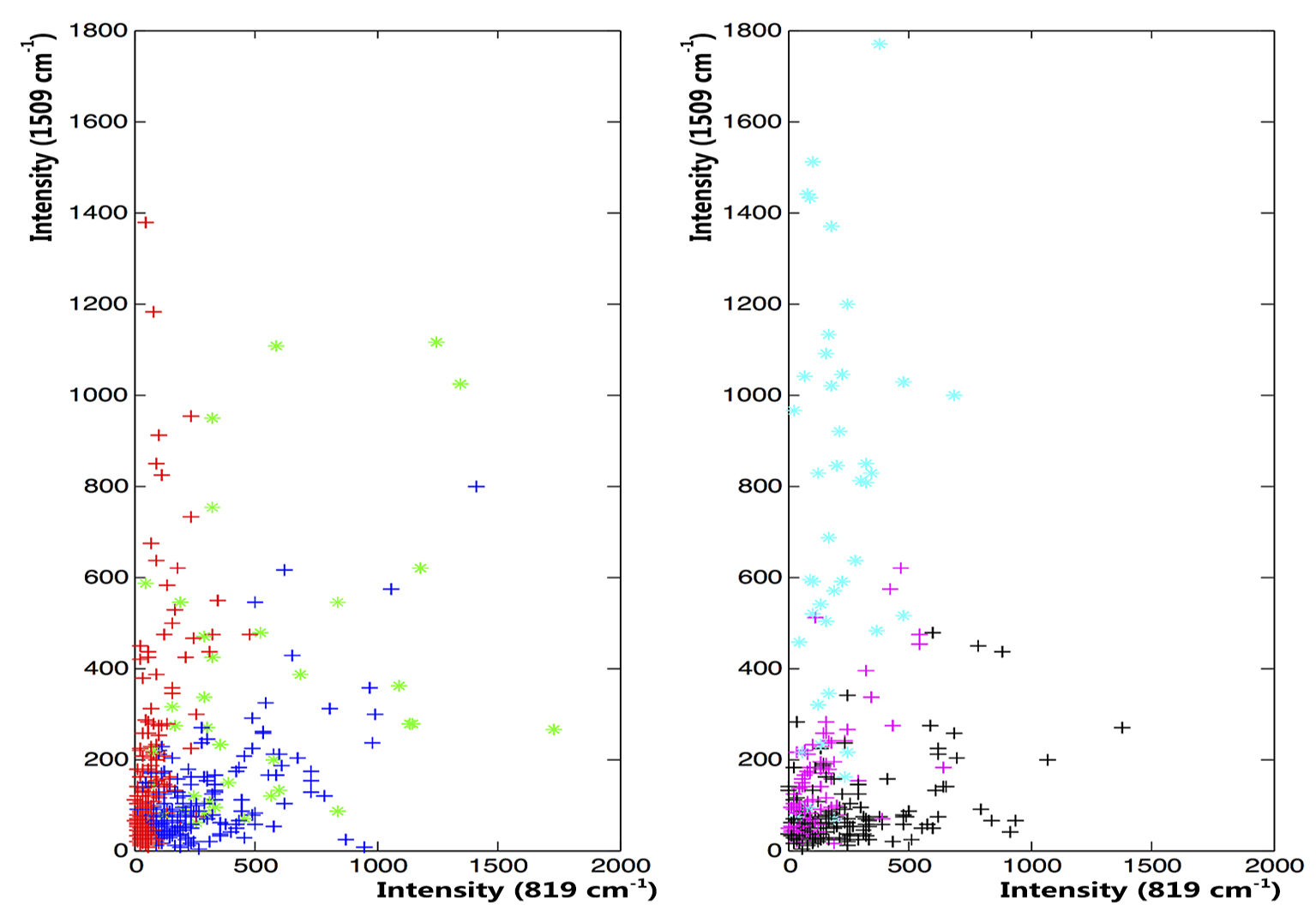


**Figure S12.** The spectral differences of categories a (red), c (blue), and e (green) in the left panel and b (black), d (pink), and f (blueness) in the right panel considering the spectral peaks at 933 (x-axis) and 1155 cm^-1^ (y-axis).


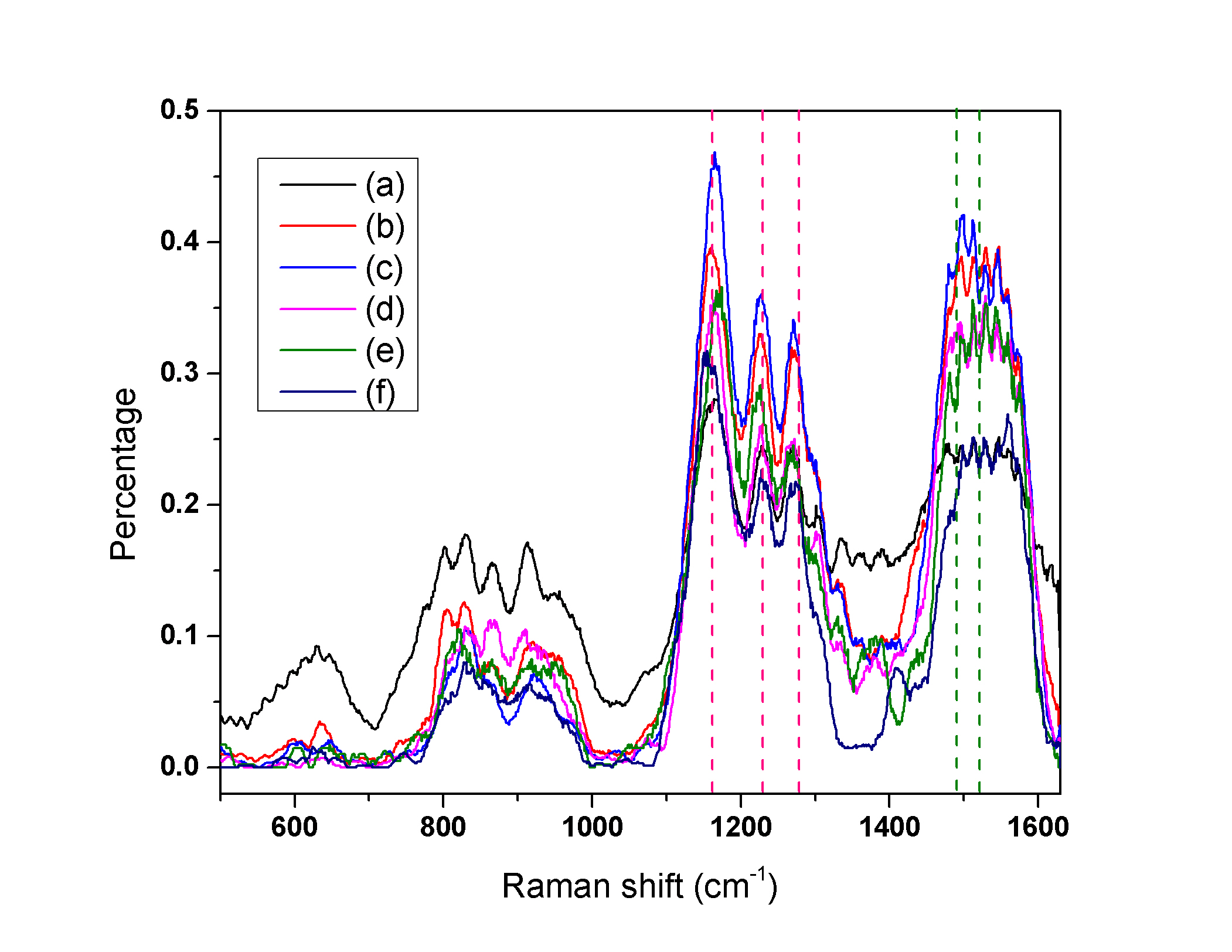


**Figure S13.** The spectral difference of the six groups defined during the apoptosis process. The curves show the probability for the observation of Raman peaks at a certain wavenumber position.
